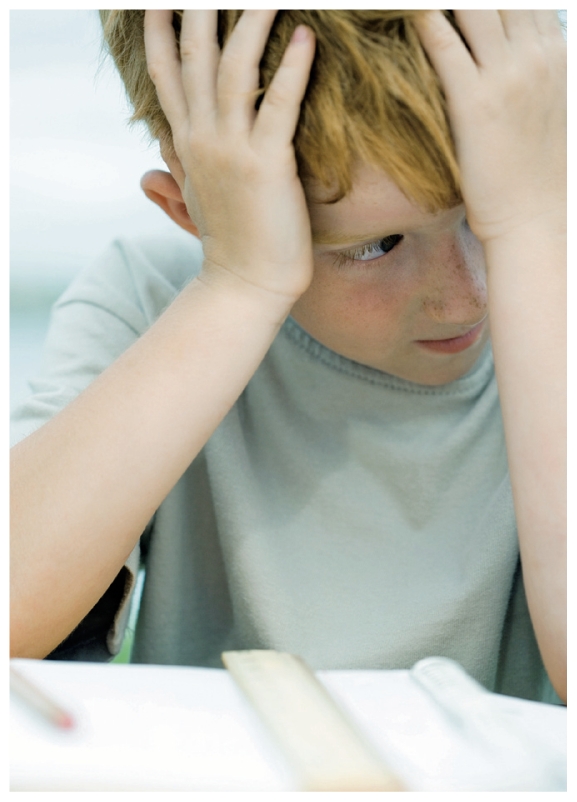# Parallel Outcomes: Comparing Effects of Environmental Contaminant Exposures with ADHD in Children

**DOI:** 10.1289/ehp.118-a542a

**Published:** 2010-12

**Authors:** Julia R. Barrett

**Affiliations:** **Julia R. Barrett**, MS, ELS, a Madison, WI–based science writer and editor, has written for *EHP* since 1996. She is a member of the National Association of Science Writers and the Board of Editors in the Life Sciences

Attention deficit/hyperactivity disorder (ADHD), the most frequently diagnosed neurobehavioral disorder in children, affects more than 5% of the population worldwide. Although the etiology of ADHD is poorly understood, characteristics similar to those seen in children with ADHD also have been observed in children and animals exposed to lead or polychlorinated biphenyls (PCBs). Two literature reviews in this issue provide an overview of ADHD diagnostic criteria and explore the parallels between behavioral test results from children with ADHD and findings from children and laboratory animals with developmental exposure to lead or PCBs **[*EHP*** **118(12):1646–1653; Aguiar et al.;** ***EHP*** **118(12):1654–1667; Eubig et al.]**. The authors conclude that exposure to these environmental contaminants, and possibly others, may increase the prevalence of ADHD.

Deficits in executive function and attention are key characteristics of both ADHD and developmental exposure to lead and PCBs. Executive function encompasses cognitive abilities critical to goal-oriented problem solving, such as working memory, response inhibition, cognitive flexibility (the ability to mentally “switch gears”), and planning. Attention revolves around alertness (the ability to become alert and focus on a task) and vigilance (sustained alertness).

ADHD has a strong genetic component but, like other neurodevelopmental disorders, appears to involve interactions between genetic, environmental, and social factors. Neuroimaging studies in children with ADHD have revealed alterations in brain regions that control executive function and attention, such as the prefrontal cortex. Alterations in catecholamine neurotransmitter signaling also are present in children with ADHD. Such signaling is potentially susceptible to damage by environmental toxicants.

Developmental exposure to lead has been widely studied and is known to have a negative impact on cognitive function as well as on children’s behavior. Across studies, cognitive flexibility, vigilance, and alertness appear to be the functions most consistently affected by lead, but evidence also exists for negative effects on working memory, response inhibition, and planning. Several studies have reported associations between blood lead levels in children and ADHD.

In contrast, researchers are only just beginning to explore the relationship between PCB exposure and ADHD diagnosis. Developmental exposure to PCBs is known to affect cognitive functions including working memory, response inhibition, cognitive flexibility, and alertness, although there appears to be little if any impact on vigilance. Animal studies further show that both lead and PCBs can reduce dopamine (a catecholamine) signaling in the prefrontal cortex of the brain.

Although levels of lead and PCBs have declined overall in the environment and in our bodies, they remain public health issues. A better understanding of their roles in ADHD and other neurodevelopmental disorders is needed, and this knowledge could be useful in investigating other environmental agents including brominated flame retardants, bisphenol A, phthalates, organophosphate pesticides, and polyfluoroalkylated chemicals, all of which have been suggested by recent research to possibly be associated with ADHD.

## Figures and Tables

**Figure:**